# Improved meta-analysis pipeline ameliorates distinctive gene regulators of diabetic vasculopathy in human endothelial cell (hECs) RNA-Seq data

**DOI:** 10.1371/journal.pone.0293939

**Published:** 2023-11-09

**Authors:** Diksha Pandey, Onkara Perumal P.

**Affiliations:** Department of Biotechnology, National Institute of Technology, Warangal, India; Indiana University Purdue University at Indianapolis, UNITED STATES

## Abstract

Enormous gene expression data generated through next-generation sequencing (NGS) technologies are accessible to the scientific community via public repositories. The data harboured in these repositories are foundational for data integrative studies enabling large-scale data analysis whose potential is yet to be fully realized. Prudent integration of individual gene expression data i.e. RNA-Seq datasets is remarkably challenging as it encompasses an assortment and series of data analysis steps that requires to be accomplished before arriving at meaningful insights on biological interrogations. These insights are at all times latent within the data and are not usually revealed from the modest individual data analysis owing to the limited number of biological samples in individual studies. Nevertheless, a sensibly designed meta-analysis of select individual studies would not only maximize the sample size of the analysis but also significantly improves the statistical power of analysis thereby revealing the latent insights. In the present study, a custom-built meta-analysis pipeline is presented for the integration of multiple datasets from different origins. As a case study, we have tested with the integration of two relevant datasets pertaining to diabetic vasculopathy retrieved from the open source domain. We report the meta-analysis ameliorated distinctive and latent gene regulators of diabetic vasculopathy and uncovered a total of 975 i.e. 930 up-regulated and 45 down-regulated gene signatures. Further investigation revealed a subset of 14 DEGs including CTLA4, CALR, G0S2, CALCR, OMA1, and DNAJC3 as latent i.e. novel as these signatures have not been reported earlier. Moreover, downstream investigations including enrichment analysis, and protein-protein interaction (PPI) network analysis of DEGs revealed durable disease association signifying their potential as novel transcriptomic biomarkers of diabetic vasculopathy. While the meta-analysis of individual whole transcriptomic datasets for diabetic vasculopathy is exclusive to our comprehension, however, the novel meta-analysis pipeline could very well be extended to study the mechanistic links of DEGs in other disease conditions.

## Introduction

Diabetic vasculopathy is a non-trivial health concern associated with vascular dysfunction. Characteristically, diabetic vasculopathy results from the extension of the basement membrane and forfeiture of vascular cells [1]. Investigational efforts towards decoding the mechanistic links of diabetic vasculopathy would greatly augment disease stratification and prediction outcomes in clinical conditions including cardiovascular diseases, kidney failure, limb amputations, blindness, and other disease manifestations that impair the endothelial cell functions [2]. Several cost-effective next-generation sequencing (NGS) technologies, that yield huge amounts of high-throughput data in less time have been developed in recent times. The relatively low cost of data generation using these technologies has attracted further researchers in transcriptome research [3]. The application of NGS technologies for RNA-Seq. data generation has further improved both the quality and quantity of transcriptome research studies, such as differentially expressed gene signature detection among different developmental and experimental groups [4].

Platform technologies including PacBio, Solexa/Illumina, Ion Torrent’s, Roche/454, and ABI/SOLiD are widely adopted by transcriptome researchers for whole transcriptome RNA Seq. data generation. In addition, open-source software tools such as Galaxy, KNIME, BWA, insiM, SanGenix, and Bowtie continue to be a convenient choice for the successful analysis of NGS data obtained [5]. However, most tools have a manifold of steps with distinct sequential operations that enable speedup of tasks at the time of execution thereby improving the overall performance while using High Performance Computing (HPC) systems [6, 7]. The utility of these systems becomes almost crucial while implementing RNA-Seq. data analysis integration and analysis from multiple origins. Gene expression studies using high-throughput sequencing approaches have gradually become more reliant on the number of read counts from every single gene that arise from the whole transcriptome RNA-seq data libraries [8, 9].

Despite transcriptomics being a revolutionary tool in the successful identification of disease biomarkers, several obstacles continue to challenge the reliability of analysis including diverse research protocols, sample collection methods, and inconsistent data analysis tactics [10]. Notwithstanding the challenges, more recently there has been a heightened emphasis on the transcriptome profiling focused on the identification of associated genes, gene signatures, and genetic variants for diabetic vasculopathy that may be clinically significant. While advancements in NGS technologies continue to facilitate and accelerate original transcriptomic research. However, an accompanying challenge is to optimally extract the latent information that is concealed within the generated datasets. RNA-seq data could encompass either the complete set of RNA transcripts such as coding, multiple forms of non-coding RNA, gene expression for a precise developmental phase, or physiological disorder [11]. Studies on these polymeric molecules construing the functional genomic elements enable better deciphering of the molecular mechanisms of development, characterization, or quantification and accelerate disease prognosis and treatment [12]. Overall DEG profiling demonstrates the relationship between the genes and disease and thereby brings out important clues for disease pathogenesis, in that way facilitating the prediction of transcriptomic biomarkers with potential for applications in disease diagnosis and prognosis [13].

### Down-stream analysis: Enrichment analysis and network analysis

Gene interactions among correlated genes is attributed to the progression and development of multifaceted diseases including diabetic vasculopathy rather than the malfunctioning of any single gene. So, analyzing such gene interaction networks, and identifying network hub genes becomes crucial for segregating, disease monitoring, and progression [14]. Furthermore, network analysis circumvents the use of probabilistic measures, allowing for a more direct identification. For discerning the disease progression patterns and pathways, several gene-based bioinformatics methodologies have been used, comprising interacting genes, proteins encrypted by genes, and network analysis [15].

A custom-built meta-analysis pipeline had been adapted for the association of DEGs from individual studies pertaining to diabetic vasculopathy. Further, functional annotation and pathway enrichment analysis of the novel DEGs were performed employing Gene Ontology (GO) and the Kyoto Encyclopedia of Genes and Genomes (KEGG). Subsequently, the mechanistic links of diabetic vasculopathy occurrence and progression were analyzed at the molecular level using protein-protein interactions (PPI) and network analysis using Cytoscape software (ClueGo plugin) to spot the significant hub genes. Visualization of the significant DEG networks has revealed potent hub genes that further reinforce our comprehension of the DEG’s biological functions and associated pathways in turn exhibiting unprecedented, distinctive, and reliable gene regulators of diabetic vasculopathy.

## Materials and methods

### Data selection and retrieval

**Fig 1:**
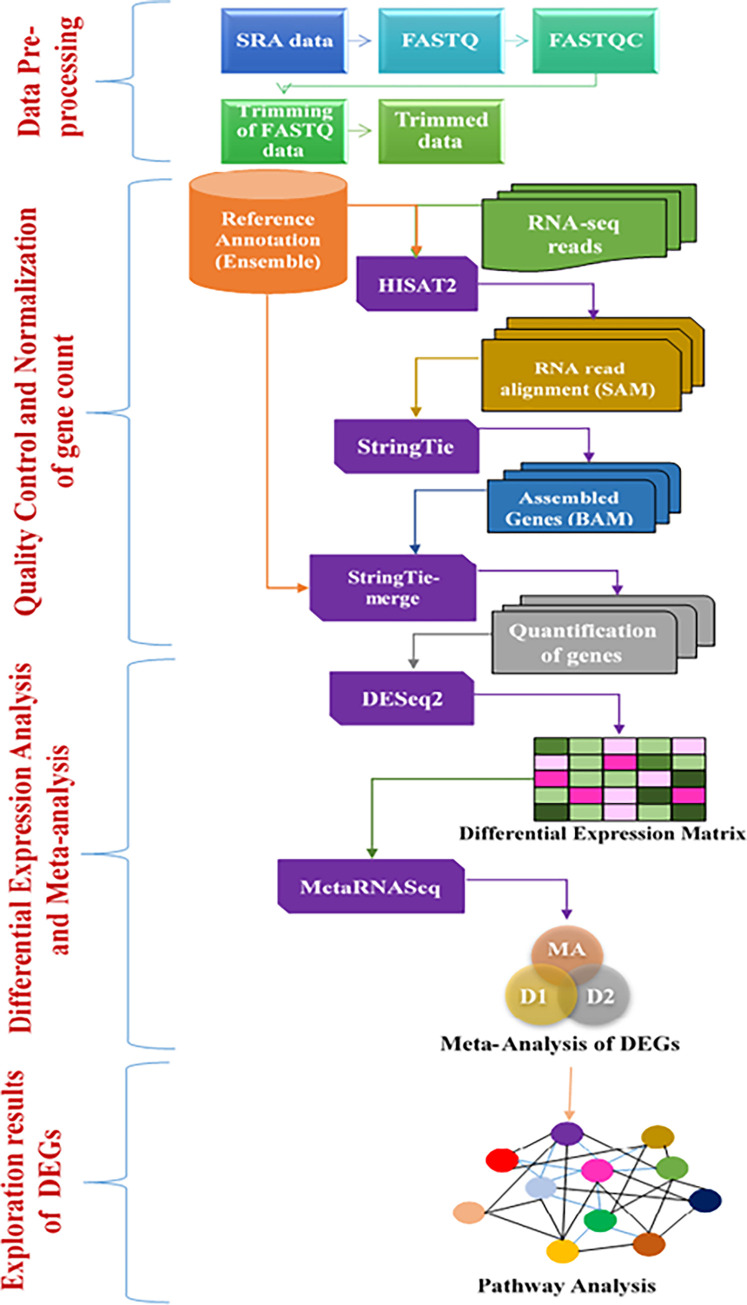
Graphical overview of integrative NGS data analysis: Pre-processing and normalization of data, identification of differentially expression genes (DEGs), meta-analysis and pathway analysis.

One of the massive functional genomics data public repository NCBI-SRA (Sequence Read Archive) database was queried for RNA-Seq studies relating to diabetic vasculopathy. The clinical statistics of FASTQ files of control and case were downloaded from the SRA database (https://www.ncbi.nlm.nih.gov/Traces/sra/) and have been described accordingly SRP095512_1 [16] and SRP092491_2 (Table 1) [17]. These only studies were related to endothelial cell samples which endothelial cell-pericyte communication leads to diabetic vasculopathy that has been obtained from human blood vessels and also possessed ample statistical power to detect significant variations in the meta-analysis. FastQC was adopted to warrant the quality of raw read sequences, low-quality bases, and adapters were discarded with a cut-off criteria (phred quality score <10), using Cutadapt_1.15–1 in the Linux Environment. (https://ubuntu.pkgs.org/18.04/ubuntu-universe-amd64/cutadapt_1.15-1_all.deb.html). The overall workflow of the analysis is illustrated in (Fig 1).

**Table 1:** Sample datasets and their access information.

Datasets	Bioproject	GEO	Platform	Tissue/cell	Condition
SRP095512_1	PRJNA358470	GSE92724	Illumina HiSeq 4000	Endothelial cells from surgical samples of human skin.	4 T2DM (Type 2 Diabetic mellitus), 6 HC (Healthy Control)
SRP092491_2	PRJNA352279	GSE89475	Illumina HiSeq 4000	Endothelial cells, FACS sorted out of vascular organoids developed from differentiated iPS cells.	2 T2DM, 2 HC

### Alignment and normalization of RNA-Seq data reads

Read sequences resulting from the RNA-Seq trimmed reads require a graph FM index (GFM) [18]. The trimmed reads were then mapped with the human reference genome GRCh38 to identify their genomic positions (http://ftp.ensembl.org/pub/release-103/fasta/homo_sapiens/dna/) implementing HISAT2 (Ver.2.2.1). Subsequently, the read sequences were organized with Samtool and all the SAM files were transformed to BAM files. After mapping the reads were aligned by implementing StringTie [19] every single read was cautiously reckoned for transcripts features (GTF file of the human genome) and assembling the numerous isoforms. Read sequences below the transcriptional noise level were discarded. After implementing StringTie to assemble transcripts, the gffcompare utility was adopted to evaluate how many of the assembled transcripts fit the annotated genes, either totally or partially, and how many were utterly novel.

If genes and isoforms from one sample don’t match those in other samples, then they were assembled by combining all transcripts via StringTie’s merge function, which combines all of the genes present in each of the samples. The merged transcripts were further run for one extra phase to re-estimate the abundance features using the estimation algorithm [20]. For expression quantification of the read count matrix, only reads that were distinctively mapped were considered.

### Differentially Expressed Genes (DEGs) detection

Transcript-level data requires gene expression values across different conditions and between the samples for recognition of differentially expressed genes in RNA-Seq studies of NGS datasets [21]. Differentially expressed genes and gene count-based techniques also require normalization of the data for comparing samples and to analyze genes that can differ significantly within and across the two conditions [22].

Differential expression analysis of the two NGS datasets was performed using the DESeq2 package in the R programming Environment, parameters including p-values, False Discovery Rate (FDR)-adjusted values, and Log2 Fold Change (Log2FC) were analyzed for every gene. Transcripts with short read counts were filtered with default values of the DESeq2 package based on the average of the normalized read counts using independent function statistics. DEGs were characterized by comparing the expression levels of control and case samples. The adjusted p-values for genes were defined as the corresponding p-values following the Wald test. For obtaining a meaningful gene expression signature, an adjusted p-value of < 0.05 and a log2FC of ≥ 2 were adopted as the cut-off criteria.

### Meta-analysis of DEGs

A study-specific meta-analysis was carried out by integrating DEGs from individual studies, to find novel gene signatures that are different from the original differentially expressed gene signatures from individual studies. Meta-analysis was conducted using the MetaRNASeq [23] package in the R programming environment. Two p-value combination techniques (i) inverse normal and (ii) fisher technique were implemented for comparison and consensus. The following formula was used to combine p-values for each gene from distinct studies [24]

$$\chi_{F}^{2}-2\sum_{n=1}^{m}log(P_{n})$$

where *P*_n_ represents the sum of the p-values from the various studies. The null hypothesis in this case is that there are no variations in the differentially expressed genes across studies and that they are dispersed as a χ^2^ with 2m degrees of freedom (m denoting the number of studies) [25, 26]. Benjamini–Hochberg false discovery rate (FDR) was used and an adjusted p-value of <0.05 was deemed as statistically significant. The inverse normal combination technique involves the allocation of weights for each dataset that is being considered for meta-analysis. The Integration-driven Discovery Rate (IDRs) is the proportion of genes that originate from meta-analysis that were not originally found in any of the individual studies while still using the same statistical parameters, whereas the Integration-driven Revision Rate (IRRs) is the proportion of genes found in individual studies but not in meta-analysis [27].

### Gene ontology and pathway enrichment analysis

To decipher the biological functions of the genes represented in the meta-analysis DEG list, the genes were subjected to Gene Ontology (GO), Reactome, Wiki Pathways, and Kyoto Encyclopedia of Genes and Genomes (KEGG) pathway enrichment analysis using the gprofiler2 package in R [28]. Gprofiler2 is a gene functional classification tool that includes a collection of functional annotation methods for researchers to examine the biological functions of differentially expressed genes.

### Protein-Protein Interactions (PPI) network analysis and identification of significant candidate DEGs and pathways

PPI network construction was implemented with protein-encoding DEGs using STRING (https://string-db.org/) [29]. The newly constructed protein-protein interaction networks were then visualized using the CentiScaPe plugin of Cytoscape, hub proteins i.e. nodes with several interaction partners were critically analyzed and functional enrichment was carried out using established PPI complex network’s GO terms and KEGG pathway using ClueGO a plugin in Cytoscape.

## Results

### Analysis of individual datasets for identification of DEGs between control and case samples

**Fig 2:**
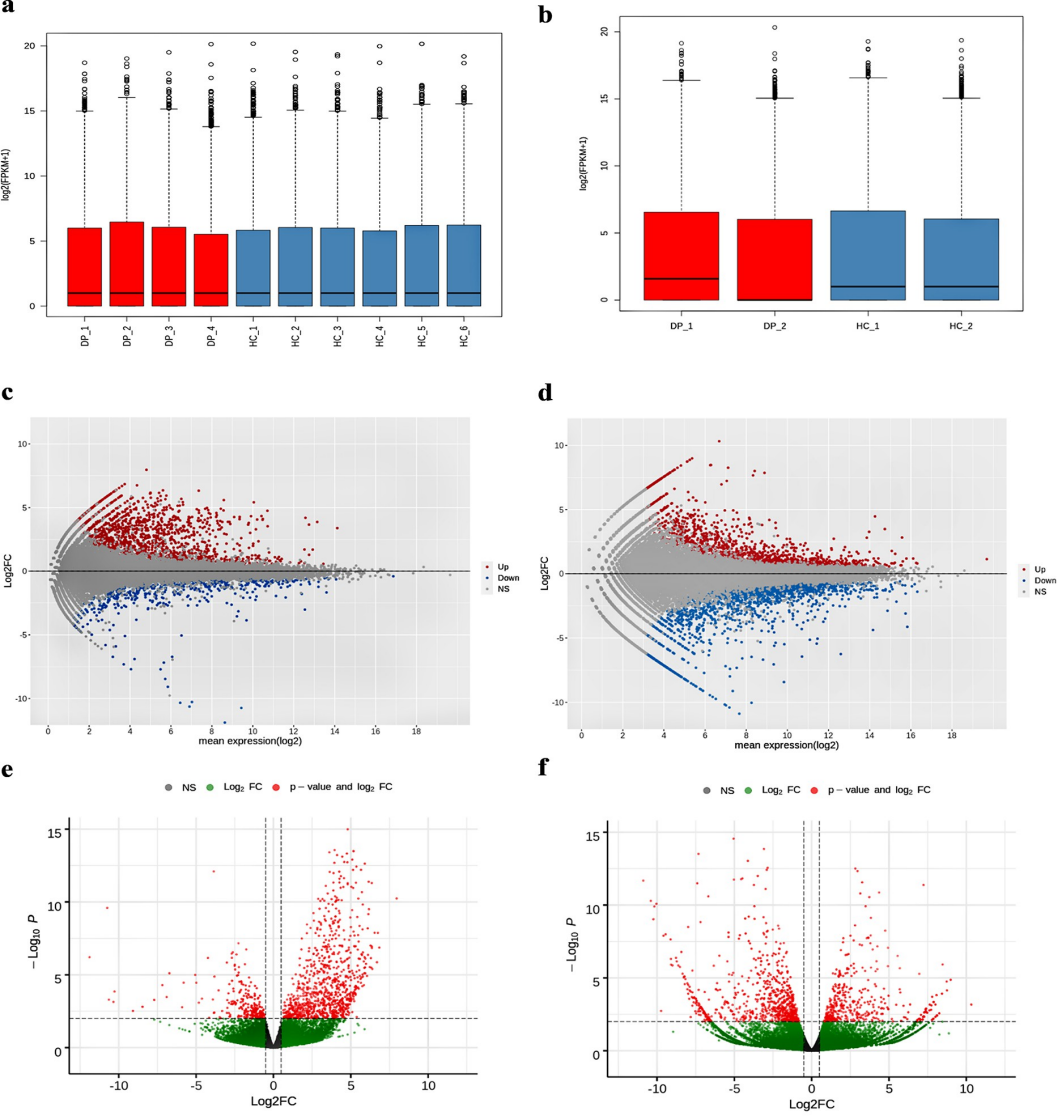
Visualization of differential expression analysis of two different RNA-studies. (a &b) Box and Whisker plot using boxplot function displaying the distribution towards median of the DEGs in RNA-Seq study1 and study2. (c & d) MA plot exhibiting the log fold-change equated to average expression designed with the ggmaplot function, with base-2 log fold-change thresholds of −1 and +1 as default exposing Up and Down regulated genes. (e & f) Volcano plot created by the enhanced volcano function, shows p-value threshold of 0.05 versus log fold-change thresholds of −1 and +1, identify genes with large fold changes which are statistically significant.

Analysis of individual RNA-Seq studies was examined using DESeq2 [30]. A total of 1130 up-regulated and 1091 down-regulated genes were identified as DEGs from dataset 1 (SRP095512_1) (Supplementary Tables ST-1, ST-2 in S1 Data) similarly a total of 2685 up-regulated and 2053 down-regulated genes, were identified as DEGs from dataset 2 (SRP092491_2) (Supplementary Tables ST-3, ST-4 in S1 Data). The up and down-regulated genes are underscored in Fig 2. The dysregulations were detected for the prescribed criteria of p-value ≤ 0.05, padj ≤ 0.002, and a log2fold change ≥ 2 respectively.

The box and whisker plots exhibited in Fig 2A & 2B are a convenient option for visualization of RNA-Seq read distribution of all samples in the datasets. As perceived from the plots the entire samples are bifurcated into control and case samples represented in blue and red colors respectively and their gene expression units are indicated as RPM (Reads per million mapped reads). If the distribution of RNA-Seq read count valuations tends to be skewed, data transformation could be applied to normalize it [31, 32]. In such case, normalized expression units become essential to eliminate technical biases in sequenced data and allow gene expressions directly comparable between and within samples. Normalization data points and statistical variables such as quartiles, median, minimum, and maximum values enable a check for outliers. Correspondingly the MA plots exhibited in Fig 2C & 2D present the log fold-change equated with average expression as a dispersion plot, with log base 2-fold-change on the y-axis, over the average of normalized read counts on the x-axis. As spotted from the MA plots, the data points spread out forming a waving effect starting from the right and progressing towards the left. For an improved, differentiated view of the data points, the ggmaplot package was implemented with padj ≤ 0.002, the up and down-regulated genes are represented in red and blue colors respectively. To expand the statistical significance and to minimize the non-significant genes between and across the samples in the datasets, Volcano plots were applied. The volcano plots displayed in Fig 2E & 2F consist of clusters of data points representing the differential gene expression patterns. Each grey dot represents an in-significant gene expression. For both RNA-Seq datasets 1 and 2, the up and downregulated genes are presented on the right and left panel respectively. In comparison, it turns out that there were a greater number of up-regulated genes for the RNA-seq study compared with study 2.

### Meta-analysis of individual datasets for identification of DEGs between control and case samples

**Fig 3:**
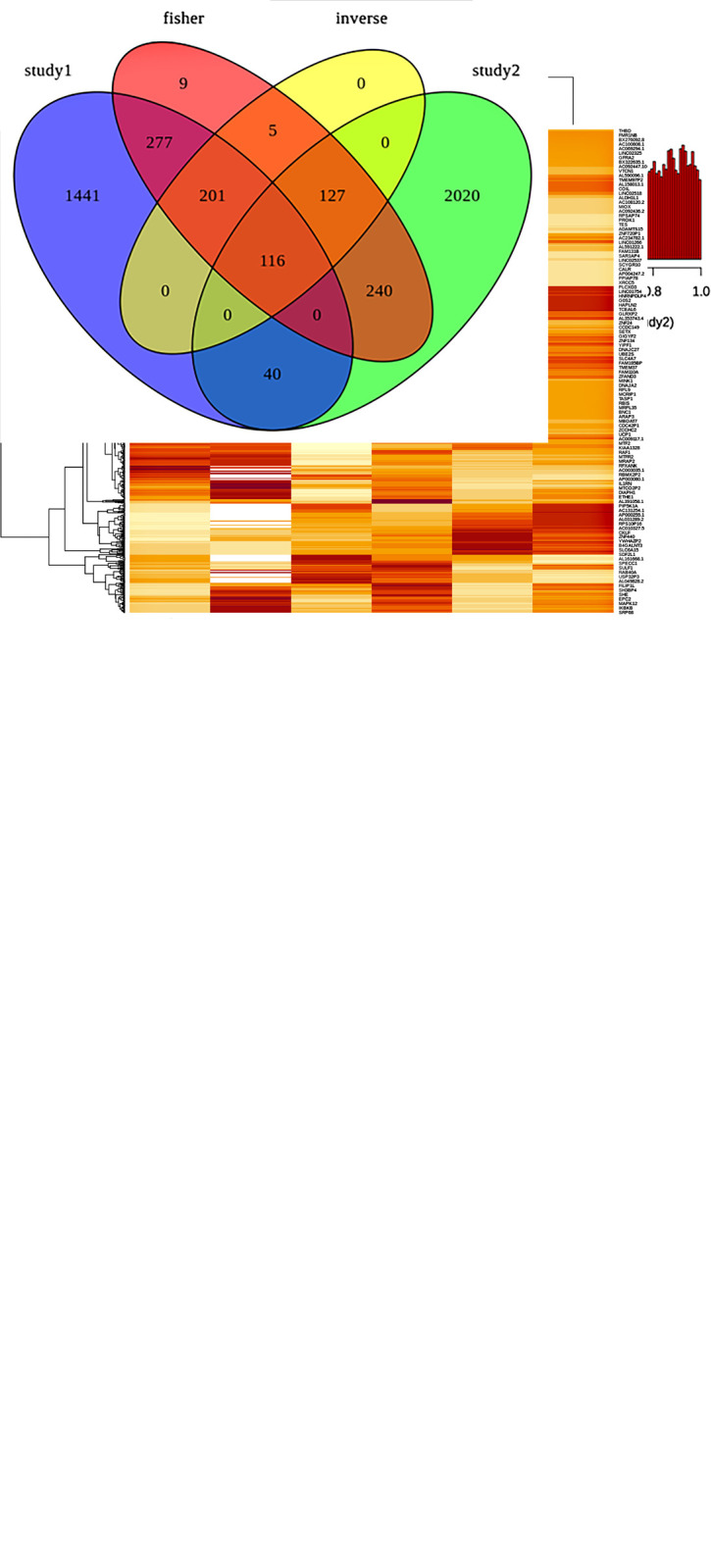
a. Histograms illustrating raw p-values from the meta-analysis attained by Fisher and Inverse normal test procedures Fisher (left) and Inverse normal (right). b. An all-inclusive Venn diagram depicting the outcomes of the differential expression analysis including the intersection of individual analysis (study1 and study 2) and the two meta-analysis methods (Fisher and Inverse normal). c. Heatmap showing the comparative expression of the 975 most significant DEGs based on the meta-analysis, where 930 genes were up-regulated and 45 genes were down-regulated (T2DM versus control). Each DEG was normalized and clustered based on the condition (T2DM vs controls) and the original dataset of each sample in the heatmap. The heatmaps were created using ggplot2’s heatmap function with Euclidean distance index, and genes with p-values >0.05 in the DESeq2 analysis were considered significant.

Meta-analysis of individual datasets for identification of DEGs between control and case samples: was conducted using metaRNASeq by combining the p-values of the two datasets. As metaRNASeq had been adapted for identifying consistently regulated genes across a variety of experiments, we have adapted the metaRNASeq method, as it is one of the most robust and reliable methods for conducting meta-analysis. A total of 68,981 gene expression levels were considered for the investigation. Two prevalent meta-analysis methods namely the Fisher [33] and Inverse Normal [34] methods were implemented. The Fishercomb function in the metaRNASeq package was adopted to combine p-values via the Fisher method and the p-values derived from the meta-analysis were subsequently scrutinized. Eight replicate weights were selected for the Inverse normal combination function for the simulation of p-values in each study. Histograms illustrating raw p-values (range 0.0 to 1.0) from the meta-analysis attained by Fisher and inverse normal test procedures are presented in Fig 3A. The frequency distribution of raw p-values shows how it widely spread across the mentioned range and its trend.

In terms of statistical significance, a large number of DEGs were found to be consistent across RNA-Seq studies. Every solitary DEGs i.e. mutually shared between and across samples from distinct studies along with the two meta-analysis methods adopted are depicted in an all-inclusive Venn diagram in Fig 3B. Out of the total number of genes considered, 975 gene expressions i.e. 930 up-regulated and 45 down-regulated were identified as significantly differential expressed in the meta-analysis employing both the Fisher and Inverse normal methods. Amid 975 genes by meta-analysis, 594 are the genes found by both meta-analysis and study1 whereas 243 genes by meta-analysis and study2. Consistent with the outcome, common 116 DEGs were reported by meta-analysis (Fisher and Inverse normal) and both individual studies. The meta-analysis criteria included parameters such as false discovery rate (FDR) correction with Benjamini-Hochberg (BH) as 0.05. (Supplementary Table ST-5 in S1 Data). Amongst the 975 gene expressions considered i.e. 930 up-regulated and 45 down-regulated DEGs, 14 genes were newly disclosed with the aid of meta-analysis in which 5 genes are common in both Fisher and Inverse normal methods, intriguingly these 14 significant DEGs were hidden and undiscovered in the reanalysis of individual differential gene expression investigations in Table 2.

**Table 2:** List of differentially expressed gene signatures newly discovered with the aid of meta-analysis methods of RNA-Seq datasets within and across control and case samples.

DEG_Ids	Gene_name	Gene depiction	BaseMean	Log2FC	Adj.P-value
**ENSG00000123689**	G0S2	G0/G1 switch 2	1.31E+00	1.06E+00	5.72E-01
**ENSG00000082701**	GSK3B	Glycogen synthase kinase 3 beta	3.89E+00	1.18E+00	4.21E-01
**ENSG00000254647**	INS	Insulin	2.68E+00	1.27E+00	1.12E-01
**ENSG00000163599**	CTLA4	CytotoxicT-lymphocyte associated protein 4	2.47E+00	1.39E+00	6.10E-01
**ENSG00000004948**	CALCR	Calcitonin receptor	4.21E+00	1.48E+00	3.83E-01
**ENSG00000162600**	OMA1	OMA1 zinc metallopeptidase	5.73E+00	1.57E+01	4.37E-07
**ENSG00000179218**	CALR	Calreticulin	-2.19E+00	1.60E+00	5.99E-01
**ENSG00000127022**	CANX	Calnexin	-3.55E-02	1.80E+03	9.35E-01
**ENSG00000115524**	SF3B1	Splicing factor 3b subunit 1	1.80E-01	1.81E+03	5.88E-01
**ENSG00000171105**	INSR	Insulin receptor	-3.98E-02	1.97E+01	9.16E-01
**ENSG00000087274**	ADD1	Adducin 1	-2.90E-01	2.09E+03	2.45E-01
**ENSG00000104365**	IKBKB	Inhibitor of nuclear factor kappa B kinase subunit beta	-5.87E-01	2.22E+01	2.94E-01
**ENSG00000131459**	GFPT2	Glutamine-fructose-6-phosphate transaminase 2	4.48E+00	4.07E+00	7.32E-04
**ENSG00000102580**	DNAJC3	DnaJ heat shock protein family (Hsp40) member C3	2.91E-01	6.89E+01	7.32E-01

These newly 14 potent genes are used to find molecular mechanisms for diabetic vasculopathy and further used in pathway analysis. In addition, every single differentially expressed gene signature identified in the meta-analysis including trends of up-or down-regulation are displayed graphically in the form of heatmap in Fig 3C respectively. To build associations between genes, the left portion of the heatmap displayed the molecular connection among them. A gene’s expression pattern is clustered with others with a similar pattern. Midst genes some are CALR, NOS3, GSK3B, PTPN11, INSR, CTLA4, GOS2, DNAJC3, INS and ATF6.

### Downstream enrichment analysis

**Fig 4:**
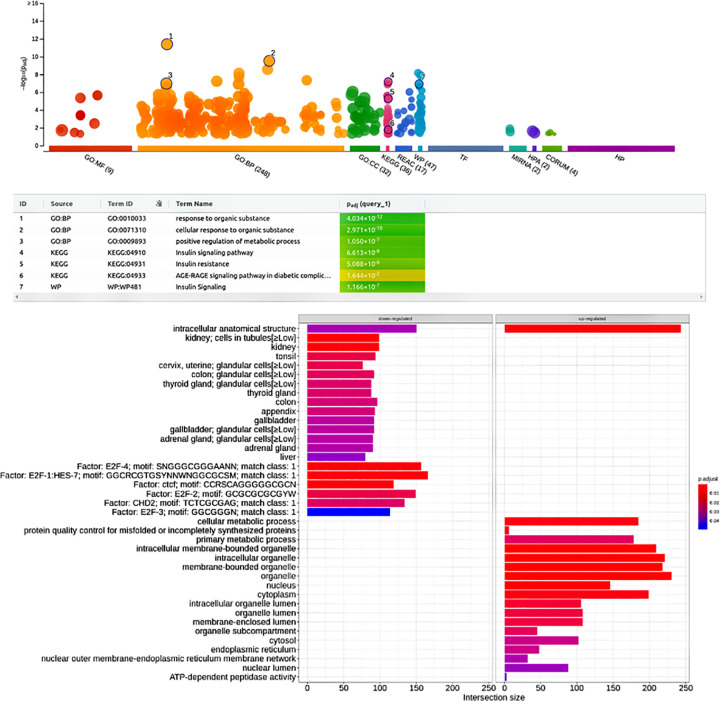
G:profiler and enrichplot enrichment outcomes from Manhattan plots. (a) Functional enrichment analysis of the 975 DEGs between T2DM and healthy control using g:Profiler, where the x-axis represents the functional terms organized according to the color codes of the source databases, while the y-axis represents the enrichment adjusted p-values in negative decimal logarithm scale. (b) An analysis of KEGG pathway of up and down-regulated gene signatures, where x-axis labels corresponding to gene ratio and y-axis labels corresponding to KEGG pathways of up and down-regulated genes. According to the bar’s color and size, the pathway has an enrichment significance level and quantity of enriched input genes.

To reveal the functional classifications of all 975 DEGs from meta-analysis, enrichment analysis of all 975 differentially expressed gene signatures was implemented using gprofiler2 and enrichplot [35] packages in the R programming environment. Upon enrichment 930 differentially expressed gene signatures were revealed to be up-regulated and 45 as down-regulated. As presented in Fig 4A and (Supplementary Table ST-6 in S1 Data), the x-axis represents functional terms, while the y-axis displays p-values corresponding to enrichment in a negative log10 scale. Each circle in the plot represents an individual functional term that is color-coded based on DEGs and scaled according to the number of genes that are annotated for that specific term. The DEGs emphasize the term groups belonging to either one of the gene ontologies, i.e. biological processes that enriched cellular response to an organic substance, activation of metabolic processes, regulation of insulin receptor signaling pathways, glycogen biosynthetic process, cellular response to insulin stimulus and TOR signaling.

### KEGG pathway analysis

As a means of finding which DEGs are triggered and suppressed in the various pathways, gene expression data was mapped to the Kyoto Encyclopedia of Genes and Genomes (KEGG). The enriched differentially expressed gene signatures were subjected to Kyoto Encyclopedia of Genes and Genomes (KEGG) pathway analysis, the analysis revealed the DEGs were primarily related to insulin resistance, type 2 diabetes mellitus, diabetic complications, AGE-RAGE signaling, and Rap1 signaling pathways. When exploring the enrichments for the set of consistently up-regulated/down-regulated genes in Fig 4B, we found enrichment in cellular and primary metabolic process, intracellular organelle, and ATP-dependent peptidase activity primarily associated with up-regulated DEGs by contrast the down-regulated DEGs were linked processes related to thyroid hormone signaling pathway, longevity regulating pathway, carbohydrate derivative binding and secretory granule.

### Protein-protein interaction network—identification of hub genes via network analysis

**Fig 5:**
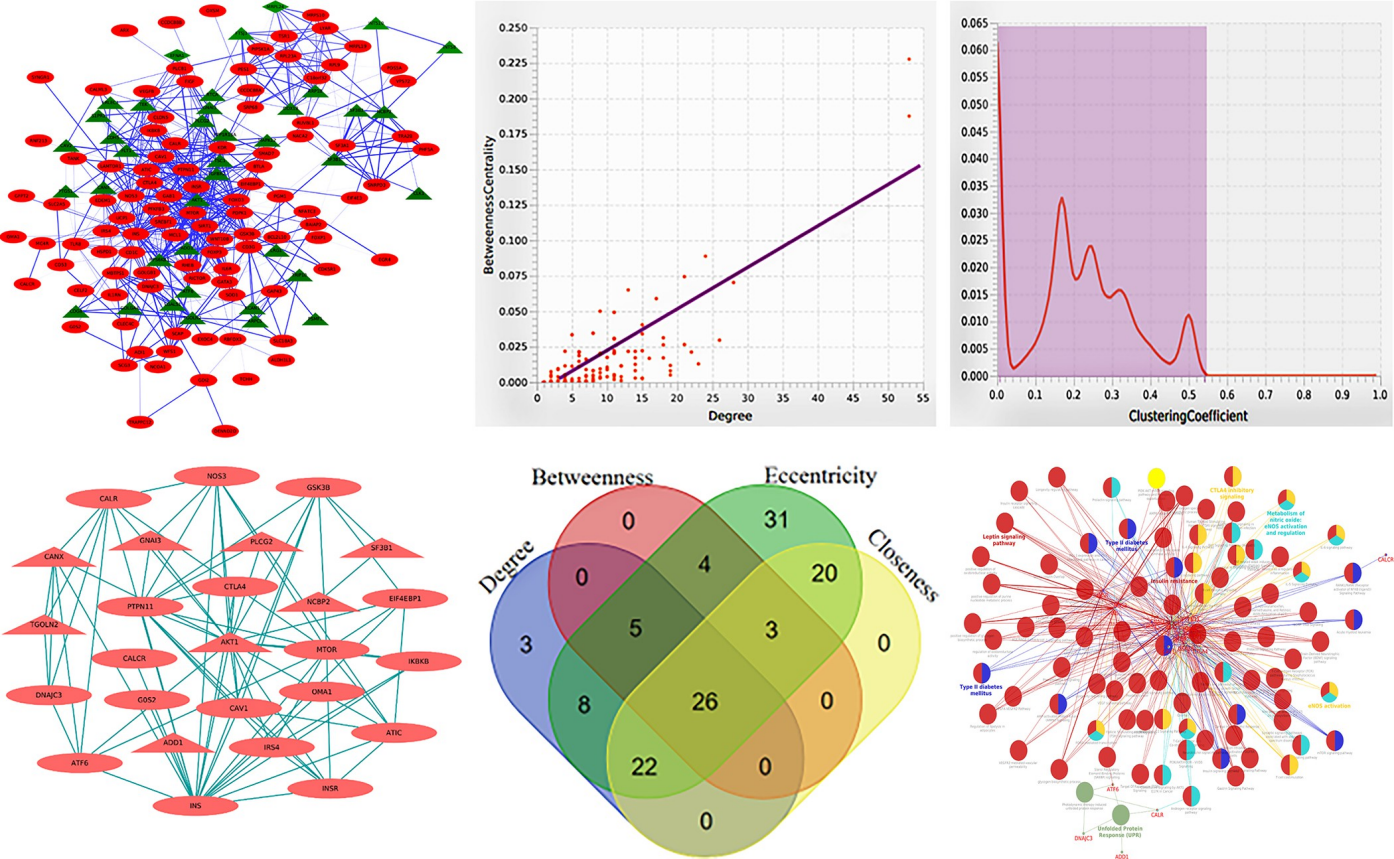
Pathway analysis encompassing the top 150 meta-DEGs: (a) Representative network model of interactions across up-regulated and down-regulated genes unravel their role in metabolic and biosynthetic processes respectively. Node colour reflects up-regulation (red ellipses) or down-regulation (green triangles). Interactions are represented by edges, with thicker edges indicating a more significant interaction. The degree distributions of nodes shown are derived on the undirected network of protein-protein interactions. (b &c) The two figures represent data distribution amongst degree versus betweenness-centrality (left) and clustering coefficient versus betweenness-centrality (right). The red line reflects an altered power rule indicating that the network under investigation is scale-free. Protein-protein interactions (PPI) among the 26 DEGs genes: (d) 26 of 150 hub DEGs (18 up-regulated genes (red ellipses), 8 down-regulated genes (red triangles) were premeditated for protein-protein interaction network complex. (e) Venn scheme depicting the intersection of four parameters intended for categorizing the hub genes. The four parameters are represented by assorted coloured regions. The overlapping regions represent the most frequently composed DEGs. The figures in each segment of the Venn scheme denotes the number of up and down-regulated genes respectively. f. Enriched pathway and hub genes disclosed with the aid of ClueGO plugin in Cytoscape: ClueGO plugin offers a comprehensive enrichment analysis for DEGs, including Gene Ontology biological process and KEGG pathways. With a kappa score of 0.4, the network’s connectivity is represented by functional nodes and edges shared by the hub genes. Only significant (i.e. p-value ≤ 0.05) pathways are emphasized in the enrichment. The node size is indicated by the p-value and represent discrete biological process. The colour code of a node displays the functional class in which it is active. Nodes of the same colour belong to the same functional group. Colour that are assorted fit into a diversity of categories. The numerous molecular pathways involved in the enrichment analysis of the known hub genes are represented by unique colour. The bold fonts indicate most significant functional pathways that determine the terms of each group’s signalling pathways. Hub genes linked in each group are highlighted in red font.

A putative PPI network with 150 nodes and 610 interactions was created using the STRING database, with a minimum required interaction score of >0.7 (high confidence) and with a PPI enrichment p-value<1.0e-16. To perceive the molecular networks in the STRING database, the stringApp plugin was employed via Cytoscape [36] (Fig 5A). Following detailed network examination every single node was sorted according to their degree value as degree distribution enables inspection of the closeness of the genes found in these networks as compared to the entire human interactome. Fig 5A illustrates each single node that could be clearly distinguished from the rest determined by their degree value.

Subsequently, additional network properties including distribution of node centralities (a revered method for identifying key nodes) were investigated by assessing parameters such as degree, betweenness, and clustering coefficient employing Network Analyzer [37]. As portrayed in (Fig 5B & 5C) the network’s degrees and clustering coefficient are represented on the x-axis, while the frequency of nodes with a given degree and betweenness are denoted on the y-axis.

Fitting of the data points was accomplished by adopting the least square approach intended for linear regression. In order to find a fitting, cut-off, and define the crucial nodes, insignificant data points from the network were discarded. As a result, 26 nodes were identified as significant considering the four parameters namely (Betweenness, Closeness, Degree, and EcCentricity) CentiScaPe network analyzer. (Fig 5D and Supplementary Table ST-7 in S1 Data).

Furthermore, we calculated the intersections of the four parameters and created a Venn scheme to classify significant hub genes using the Venn tool (Available online via http://bioinformatics.psb.ugent.be/webtools/Venn/) (Fig 5E). Functional analysis revealed that most of these hub genes were linked to insulin signaling pathway, protein tyrosine phosphatase non-receptor, mechanistic target of rapamycin kinase, AKT serine/threonine kinase, and OMA1 zinc metallopeptidase. Among the illustrated genes, the DEGs that altered the metabolic behaviour of the signaling pathways were linked closely to altered vascular permeability thereby contributing to the progression of complications such as coronary artery disease and cardiovascular disease.

In essence, the results observed from gene ontology biological process and KEGG pathway analysis included longevity regulating pathway, insulin resistance, insulin signaling pathway, CTLA4 inhibitory signaling, metabolism of nitric oxide, and leptin signaling pathway, which might be befitting for the hub gene signaling pathways displayed in (Fig 5F and Supplementary Table ST-8 in S1 Data).

## Discussion

In coherence with our hypothesis, the focus of the present study was principally on the investigation of novel gene regulators in diabetic vasculopathy in humans [38].

While prior studies have adapted NGS data analysis for diabetic vasculopathy investigations and have established several mechanistic links including the role of mRNA-influenced endothelial inflammation through modulating interleukin-1 receptor-associated kinase-1 (IRAK-1) in diabetic vasculopathy [39], hyperglycaemia-induced hypomethylation of promoter region of RNA, which is also a common occurrence in diabetic vasculopathy [40] identifying factors specific to chronic diabetic microangiopathies, to identify genes and risk factors in diabetic vascular organoids [41]. Use of co-culture techniques to identify diabetic vasculopathy networks using iPSC-derived endothelial cells and pericytes [42] self-assembly of human pericytes and endothelial cells in capillary networks that are encased by basement membrane in blood vessel organoids. Nevertheless, in this study, a quantitative meta-analysis has been implemented adopting two individual RNA-Seq data studies that have focused on investigating unique human vascular tissue models to profile regulators of gene expression and novel genes linked to type 2 diabetes mellitus.

In order to improve the overall data integration of the RNA-Seq data from diverse studies the raw data (in FASTQ format) from the individual studies, were subjected to stringent quality control and normalization protocols to ascertain data consistency (Fig 1) [43].

While it is apparent that commercial NGS technologies employ proprietary platform-specific standards and formats. For example, the Illumina next-generation sequencing (NGS) platforms generate about 25–250 nucleotide sequence reads that are stored in FASTQ format [44] it becomes necessary that the reads be prudently trimmed while reviewing RNA reads to avoid dire consequences in the steps that trail the pre-processing stage of trimming. So, we adopted a lenient trimming approach as a middle ground, thereupon retaining biological information that was not reported optimally while concurrently discarding insignificant data. As inaccurate pre-processing of raw data impacts the overall mapping process and results in low mapping frequencies, for all the mapped reads against the human genome we ensured that the estimated mapping frequency of RNA-Seq read counts is anywhere between 70% and 90%; nevertheless, when such reads were mapped against the transcriptome, the expected mapping frequency was always slightly lower [22].

About 68,981 gene signatures were considered (Supplementary Tables ST-1‒ST-4 in S1 Data) for the quantification of gene expression analysis adopting DESeq2. DESeq2 generates statistically significant outcomes by incorporating precursive distributions to estimate dispersions and logarithmic fold changes. Moreover, DESeq2 is proficient in shrinkage log fold-change towards zero-centred for stable estimations and noise removal, signifying that expression values that have relatively lower averages have higher log fold-change variability than expression values with relatively higher average values [45]. Eventually, a subset of 975 gene signatures including 930 up-regulated and 45 down-regulated gene signatures (Supplementary Table ST-5 in S1 Data) were predicted as differentially expressed.

Rather than using transcript values as such for the meta-analysis, we chose the Wald test [46], a well-established method derived from the amalgamation of one-sided z-value for the independent studies that are evaluated from the p-values attained by DESeq2, owing to the negative transformation of p-values, the larger the y-axis, the lesser the p-value. Differentially expressed genes between control and case datasets had identified an enormous transcriptomic response in both studies. Even though certain DEGs overlapped between the two RNA-Seq reports, a quantitative meta-analysis was performed adopting a combination of methods namely inverse normal and fisher methods implementing MetaRNASeq [23] to improve the acceptability of the DEGs analysis. Meta-analysis is a method employed to integrate data. In its fundamental application, it is utilized to consolidate outcomes from various independent tests that relate to the overarching hypothesis. Nonetheless, prior statistical approaches for aggregating studies (Keita Tamura et al., 2022, Zixun Yuan et al., 2022 and Kakeru Yokoi et al., 2022) involving RNA-seq data in humans employed traditional differential expression analysis and did not prioritize the prediction of anti-endurance genes. Instead, their primary focus was on identifying genes that exhibited tendencies toward overexpression or under-expression. The reported pipeline utilized distinct methodologies from the meta-analysis approach, making direct comparisons unfeasible. However, similar to all meta-analyses, the p-value combination techniques described here need to address inequality in experimental goals, designs, and target populations, in addition to variations in library preparation, sequencing technology, and lab-specific influences [47–49].

While the overall number of relevant under-expressed gene signatures significantly exceeded the number of over-expressed gene signatures in the DEGs meta-analysis, a subset of 116 gene signatures were found to be common among the individual analysis and the meta-analysis (Fig 3B & Table 1). Remarkably, 14 DEGs were undiscovered in individual analysis but revealed only in the meta-analysis. We presume that these novel DEG signatures from the meta-analysis advocate the existence of heterogeneity in type 2 diabetes mellitus-associated gene expression changes in vascular permeability of endothelial cells [16], which are consistent with prior findings using pathway enrichment analysis and protein-protein interactions performed employing RNA studies [50].

Amidst the subset of DEGs, the prominent gene signatures include INS, INSR, DNAJC3, OMA1, GOS2, and CALR which have been reported to be differentially expressed in type 2 diabetes mellitus and/ or metabolic disorders. INS encodes for insulin, which stimulates the proliferation of vascular smooth muscle cells (VSMC), of specific interest is diabetic resistance vessels have revealed significant abnormalities [51–53]. INSR, encode insulin receptor that are responsible for accelerated atherosclerosis within vascular smooth muscle cells in diabetic patients [54]. DNAJC3 is another significantly upregulated gene in the endothelium of renal arteries in diabetic patients due to AGEs and oxidized low-density lipoproteins being exposed to endothelial cells [55]. OMA1 plays a central role in inducing prothrombotic and proinflammatory cytokines and growth factors, which lead to diabetic vasculopathy [56]. Although encoded in the nuclear DNA GOS2, is connected to oxidative phosphorylation and eventually results in an increase of mitochondrial gene expression in diabetic gastroenteropathy [57]. Especially in skeletal muscle and mitochondrial samples, CALR was consistently detected as a valuable indicator of mitochondrial function associated with type 2 diabetes mellitus [58]. Lastly, GFPT2 gene variations (glutamate and /or aspartate mutants) were shown to be associated with type 2 diabetes mellitus [59]. While prior studies have reported similar biomarker identification approaches, these studies have implemented altogether different techniques. For instance (R. A. Wimmer *et al*., 2019), have analyzed the expansion of self-organizing human blood vessel organoids from pluripotent stem cells eventually reporting the key drivers of DLL4 and NOTCH3 in human blood vessels [16]. Likewise, (M. V. González *et al*., 2020) have generated induced pluripotent stem cells (iPSCs) with full responders and non-responders, demonstrating prolonged and marked significant variations [17]. However, both studies have been accomplished by adopting an *in-vitro* rather than *in-silico* approach which limits interpretation of the study outcomes using direct comparison. Although at the individual level, *in-vitro* studies are advantageous, inherently *in-silico* meta-analysis approaches are efficient in data integration especially heterogeneous data of multiple origins. Also, novel genes revealed via *in-silico* meta-analysis greatly complement the results of *in-vitro s*tudies and *vice versa*.

To discern in what manner these DEGs, dispense with the progression of vasculopathy, supplemental functional inquiries were implemented. We performed an integrated pathway analysis on these genes by implementing Gene Ontology (GO) and KEGG databases [60]. Predictably the DEGs were strongly associated, implying that they are co-expressed with a vast number of genes in each group and contribute to a variety of biological processes. An exhaustive GO biological process has revealed that the significant DEGs were predominantly associated with glycogen biosynthesis, extracellular matrix reorganization within the endothelial cell function, and also in disrupted endothelial cell-pericyte communication [61]. Concerning GO molecular function, the up-regulated DEG signatures were significantly enriched in growth factor binding such as insulin receptor binding and carbohydrate derivative binding as far as down-regulated DEG signatures were concerned these DEGs were significantly enriched in hormone binding, vascular endothelial growth factor, and transcription factor binding portraying a vital role in the occurrence and progression of type 2 diabetes mellitus vasculopathy [62]. Meanwhile, KEGG pathway analysis exhibited that the DEGs were significantly enriched in the insulin signaling pathway, AGE-RAGE signaling pathway, and type 2 diabetes mellitus [63]. Consequently, biological process, molecular function, and pathway enrichment analysis revealed a robust association of DEGs with the cellular metabolic process, establishing a downright connection with insulin resistance.

In an attempt to comprehend the PPI network and to visualize the associations and interactions between the top 150 DEG signatures, we have resorted to Cytoscape (Fig 5A). The molecular interaction networks cataloguing factor revealed 26 DEGs as potent hub proteins also referred to as bottleneck scorer proteins. These 26 significant proteins (CALR, NOS3, GSK3B, PTPN11, INSR, CTLA4, EIF4EBP1, DNAJC3, INS, ATF6, CALCR, IRS4, ATIC, MTOR, OMA1, IKBKB, G0S2, CAV1) were classified as up-regulated DEGs and (CANX, GNAI3, PLCG2, SF3B1, TGOLN2, AKT1, ADD1, NCBP2) were classified as down-regulated DEGs (Supplementary Table ST-8 in S1 Data), The variance in the network topology was improved by eliminating insignificant hubs with top centrality. As could be expected, quite a few DEGs were required to be evicted in this transaction. As far as the network’s topological properties are concerned, it is a scale-free network in which all nodes are unrestricted to interact globally. However, the network’s degree of distribution fits a smaller quantity of high-degree nodes and an enormous number of low-degree nodes [37].

In anticipation of prioritizing the hub genes, we took advantage of the ClueGO software. The primaries revealed that the vast majority of the biological processes and pathways correlate with diabetic vasculopathy, precisely in altered vascular permeability, tetrahydrofolate metabolic pathway, fatty acid metabolic pathway, insulin resistance, pancreatic cancer, glucagon signaling pathway, diabetic comorbidities including obesity signifying that these signaling pathways possibly are the primary contributors to the vasculopathy in type 2 diabetic mellitus [64].

## Conclusion

A factual meta-analysis pipeline for the quantitative meta-analysis of novel DEGs in diabetic vasculopathy has been presented. In comparison with DEG signature profiles originating from individual analysis of RNA-Seq datasets, our quantitative meta-analysis investigation has discovered a novel subset of DEG signatures that measure up to the mandates for latent gene regulators of diabetic vasculopathy. Additional downstream analysis has unveiled more profound mechanistic links pertaining to accompanying pathways, biological processes, molecular functions, and molecular interaction networks. For most of the novel subset of DEG signatures including CTLA4, CALR, G0S2, CALCR, OMA1, and DNAJC3 unveiled in our study, to the best of our knowledge and belief there is barely any communication that demonstrates a direct association between these novel regulators and diabetic vasculopathy. Besides, a more exhaustive large-scale study might be all the more strategic in unravelling further insights into the mechanistic links underlying the disease incidence and progression. Nevertheless, substantiating the predicted outcomes with experimental analysis in diabetic vasculopathy models draws us even closer to realizing the full potential for better management and prevention of diabetic vasculopathy and allied complications like chronic diabetic microangiopathies.

## Supporting information

S1 Data Supplementary tables (ST 1–8). (XLSX)
